# Chromatin from Peripheral Blood Mononuclear Cells as Biomarkers for Epigenetic Abnormalities in Schizophrenia

**DOI:** 10.1155/2009/409562

**Published:** 2009-07-22

**Authors:** David P. Gavin, Rajiv P. Sharma

**Affiliations:** ^1^The Psychiatric Institute, University of Illinois at Chicago, 1601 W. Taylor St., Chicago, IL 60612, USA; ^2^Department of Psychiatry, College of Medicine, University of Illinois at Chicago, 912 S. Wood St., Chicago, IL 60612, USA

## Abstract

*Background*. Studies have implicated abnormalities in epigenetic gene regulation in schizophrenia. *Presentation*. We hypothesize that identifying abnormalities in chromatin structure and the epigenetic machinery in peripheral blood mononuclear cells (PBMC) from schizophrenia patients could (a) help characterize a subset of schizophrenia patients and (b) lead to targeted pharmacological interventions. *Testing*. Investigate the relationship between clinical symptoms, demographics, hormonal fluctuations, substance abuse, disease characteristics across the major mental illnesses, and epigenetic parameters in PBMC. In addition, examine the effects of individual antipsychotics, mood stabilizers, as well as experimental agents both as clinically prescribed as well as in cultured PBMC to understand the effects of these agents on chromatin. *Implications*. If PBMC could serve as a reliable model of overall epigenetic mechanisms then this could lead to a “biomarker” approach to revealing pathological chromatin state in schizophrenia. This approach may provide an informed method for selecting chromatin modifying agents for psychiatric disorders.

## 1. Background

Chromatin conformation regulates the access and attachment of transcriptional regulators (either activators or repressors) to gene promoters. Deacetylation of histones, catalyzed by the histone deacetylase (HDAC) family of enzymes, dimethylation of lysine 9 of histone 3 (H3K9me2), and DNA hypermethylation, catalyzed by the DNA methyltransferase (DNMT) family of enzymes, are several examples of chromatin modifications that lead to a restrictive chromatin state both site specifically and globally [1], while hyperacetylation of histones 3 and 4 promote gene regulation. Because chromatin “plasticity” can be modified by conventional pharmacology, a novel approach to the regulation of gene expression in clinical populations is possible. 

Increasing evidence indicates the existence of epigenetic gene regulatory abnormalities in schizophrenia. Evidence supporting this hypothesis includes studies in which mice administered methionine were found to exhibit some of the endophenotypes of schizophrenia [2], as well as studies using animal and cell models demonstrating that the schizophrenia candidate genes the 67 kDa isoform of glutamic acid decarboxylase (GAD67) and reelin are regulated through epigenetic mechanisms [3–5]. Several postmortem studies have found an increase in the gene expression of repressive enzymes including DNMT1 and HDAC1 in schizophrenia [6–8], while others have demonstrated abnormalities in the coordination of repressive processes [9]. Finally, studies using peripheral blood mononuclear cells (PBMC) from human subjects have found reduced levels of the “open” chromatin mark, acetylated histone 3, in schizophrenia patients compared to nonpsychiatric controls or bipolar subjects [10, 11], increased levels of the “closed” chromatin mark H3K9me2 compared to controls [12], and a reduced ability to alter chromatin structure using HDAC inhibitors both as clinically administered [13] as well as in culture [10, 12]. 

## 2. Presentation of the Hypothesis

### 2.1. Understanding Epigenetic Gene Regulatory Abnormalities in Subsets of Schizophrenia Patients Using PBMC

Unlike many illnesses in which investigators and clinicians are capable of assessing the diseased tissue while the afflicted patient is still alive, psychiatry is limited by the inaccessibility of the organ of interest. In addition, representing the higher order cognitive dysfunctions present in schizophrenia using animal models is difficult if not impossible. Therefore, there exists a long history of searching for peripheral markers capable of reflecting the pathology within the brain. There are several factors which make PBMC particularly useful for serving as a model of epigenetic gene regulation in the brain. 

Firstly, previous studies have demonstrated that PBMC can provide a reliable means for studying the impact of environment/life experiences on chromatin structure and DNA methylation. Fraga et al. [14] examined several epigenetic parameters in lymphocytes from monozygotic twins at ages between 3 and 70 years old. They found that while 3-year-old twins are virtually indistinguishable in terms of their global levels of DNA methylation, acetylated histone 3, and acetylated histone 4, 50-year-old twins had significant differences on these measures. In general, older twins had greater differences in terms of epigenetic parameters than younger twins. In addition, it is important to note that these differences were consistent in subjects across at least 12 weeks, indicating that global measures of epigenetic parameters in lymphocytes are a reliable method for ascertaining chromatin state. These findings indicate that chromatin from lymphocytes may provide a “molecular fingerprint” reflective of an individual's environment, life experience, and stochastic factors which would not be revealed through genetic testing. 

Secondly, the analysis of gene regulation in nucleated blood cells from living patients undergoing the full evolution of their disorder, including response to pharmacological, metabolic and environmental events is the only foreseeable approach for prospective longitudinal clinical research, and appears to be a natural progression from single point postmortem brain studies. This is because PBMC share much of the nonsynaptic biochemical environment of neurons, such as neurohormones, neuropeptides, chemo/cytokines, metabolites, and medication blood levels. Examples of epigenetically relevant substances present in the blood that are found to have relevance to schizophrenia include methionine, which both worsens psychosis in schizophrenia and alters chromatin structure in the brain [4, 15, 16], homocysteine, which has been found to be elevated in the plasma of schizophrenia patients and is the metabolite of the DNA methyl donor S-adenosylmethionine [17–20], and valproic acid (VPA), which we have previously shown to significantly alter global chromatin structure in a dose dependent manner and affect clinical symptoms [13]. 

Finally, PBMC contain the full complement of epigenetic enzymes and machinery found in most tissues including both neurons and peripheral nucleated cells [21, 22]. Previous studies have shown that PBMC are capable of reflecting overall abnormalities in epigenetic mechanisms also thought to be present in the brain. For example, in Huntington disease, a disorder known to be associated with dysfunctions in histone acetyltransferase, a similar pattern of transcriptional repression across several chromosomes was found in blood and brain [23]. Also, several studies have shown that peripheral markers are able to discern differences in chromatin structure in twins discordant for mental illness [24–26], as well as show similarities in epigenetic parameters among individuals affected by the same illness [24, 25].

Although, PBMC may be capable of reflecting overall changes in epigenetic mechanisms they would not be appropriate for studying brain-specific processes such as synaptic plasticity and neurotransmission. Also, the expression of any one gene at a particular time in a PBMC may not be reflective of the expression of the same gene in the brain, just as the expression of a particular gene in a pyramidal cell at a given time may not be the same as that in an interneuron or glial cell. In addition, there are some disorders in which abnormal epigenetic parameters are tissue specific. For example, cancer cells have particular abnormalities that one would not expect to find in healthy tissue. Therefore, if schizophrenia is characterized by brain-specific chromatin alterations then one would not expect to see changes in PBMC. Also, global DNA methylation tissue patterns have been found to significantly differ between tissue types [27, 28]. However, the differences between individuals or diagnostic groups on global epigenetic measures have been found to be present across tissues [23]. 

We now hypothesize that PBMC may be capable of reflecting the epigenetic machinery within an individual and provide a means for discerning those subsets of schizophrenia patients who possess profound abnormalities in chromatin structure or DNA methylation. It may also help understand the impact of hormones, medications, and drugs of abuse on chromatin. Finally, it could provide a tool to both aid in the development of new chromatin altering agents as well as identify patients most likely to benefit from these types of medications. 

There is now a growing literature to support the idea that PBMC may be a useful tool for understanding the overall epigenetic machinery within an individual. In a previous study we clinically treated schizophrenia and bipolar subjects for four weeks with the only HDAC inhibitor approved for psychiatric use, VPA. Similar to animal models in which VPA was shown to increase GAD67 mRNA expression in the brain [2], we found that in subjects with clinically relevant serum levels of VPA there was a significant increase in GAD67 mRNA expression in PBMC [11]. It was our hypothesis at the time that chromatin in PBMC from schizophrenia subjects would be less responsive to VPA. This hypothesis was confirmed when it was noted that there was less of an increase in the “open” chromatin marks, acetylated histones 3 and 4 in schizophrenia subjects [13]. However, unexpectedly we also found that at baseline schizophrenia subjects had significantly lower levels of acetylated histone 3 compared to bipolar subjects [11]. Moreover, we found associations with these measures of chromatin state and symptomatology. In these preliminary analyses we found subjects with higher baseline Young Mania Rating Scale (YMRS) scores to have higher acetylated histone 3 levels and show greater increases in acetylated histone 3 after four weeks of clinical treatment. In addition, subjects with higher baseline acetylated 3 levels showed greater improvement on the YMRS as well as the total Positive and Negative Syndrome Scale (PANSS). In other words, those subjects who had the most mania symptoms were more likely to alter their chromatin in response to VPA, and those subjects with higher acetylated histone 3 levels were more likely to respond favorably to VPA treatment [11]. 

As a consequence of these studies it was decided to attempt to perform similar analyses using cultured PBMC. This model held several advantages relative to clinical treatment using HDAC inhibitors. Namely, chromatin from cultured PBMC are protected from unforeseeable or undisclosed changes in a patient's physiology such as medication noncompliance, substance use, environmental exposures such as infections and diet, hormonal fluctuations, and differences in metabolism of HDAC inhibitors. Further, the in vivo study was limited by the fact that VPA is the only known chromatin altering drug approved for use in psychiatric patients, and an in vivo comparison using chromatin altering medications to nonpsychiatric controls would not be ethical. 

Similar to the previous studies in which systemic administration of VPA was found to increase GAD67 mRNA expression in human PBMC and in brains from mice, we found comparable increases in GAD67 mRNA in human PBMC cultured with equivalent doses of VPA [11]. Also in keeping with the in vivo study schizophrenia subjects were found to have an abnormally restrictive chromatin state as indicated by lower levels of acetylated histone 3 and higher levels of the “closed” chromatin mark, H3K9me2 [10, 12]. Moreover, cultured PBMC were found to be capable of demonstrating the lack of “plasticity” in schizophrenia chromatin structure discovered in the in vivo study, in that we found less change in GAD67 mRNA expression, H3K9me2 levels, and acetylated histone 3 levels in PBMC cultured with an HDAC inhibitor compared to nonpsychiatric controls [10, 12]. Finally, we found that subjects with earlier ages of onset had higher levels of H3K9me2 [12].

## 3. Testing the Hypothesis

One means of testing the impact of particular environmental factors on chromatin would be to culture PBMC with substances such as hormones, medications, or drugs of abuse and evaluate the impact of these agents on chromatin structure. These findings could then be evaluated in light of findings from PBMC taken directly from subjects. For example, recent epidemiological evidence indicates that women may be less prone to develop schizophrenia [29], and data from PBMC cultures reveal sex differences in the ability of HDAC inhibitors to alter chromatin structure [10]. Therefore, it is a reasonable hypothesis that female sex hormones may have a protective effect in terms of schizophrenia risk through their ability to alter chromatin. The impact of sex hormones on chromatin structure could be tested by culturing PBMC with estrogen, progesterone, or testosterone. Findings from these experiments could also be compared to chromatin from animal and postmortem studies to ascertain whether similar effects are seen in other tissues.

In addition, extensive studies could be conducted both in vivo and in vitro using human subjects to examine whether schizophrenia or characteristics of schizophrenia are associated with abnormalities in epigenetic machinery in PBMC. An examination of baseline levels of epigenetic parameters, such as histone modifications, DNA methylation, and epigenetic enzyme expression could be conducted using samples from human subjects to determine whether abnormalities exist. Previous studies indicate abnormalities in the coordination of epigenetic processes in schizophrenia [9–11, 13]. Chromatin altering medications could be administered either directly to human subjects or cultured with their PBMC as molecular probes into this system. However, considering VPA is the only chromatin altering agent clinically approved in psychiatry, this greatly limits the in vivo approach. If an in vitro culture is capable of determining those schizophrenia patients with abnormalities in epigenetic gene regulation then information derived from the culture could be used to select subjects most likely to benefit from chromatin altering drugs. Once established using in vitro methods, clinical trials could be implemented whereby subjects most afflicted by abnormal epigenetic mechanisms could be treated with agents aimed at normalizing these abnormalities.

## 4. Implications of the Hypothesis

If PBMC, whether exposed to chromatin altering medications as clinically administered or through in vitro culturing, could serve as a reliable model of overall epigenetic mechanisms, then this could lead to a “biomarker” approach to revealing pathological chromatin state in schizophrenia. This approach could also provide information regarding the reaction of a subject's chromatin to medications prior to clinical treatment, thereby perhaps providing an informed method for selecting psychiatric medications for certain disorders. Finally, associating certain epigenetic abnormalities with symptoms and disease characteristics could lead to a better understanding of the etiology of symptoms with the potential for improved diagnostic validity more informed by a patient's biology.
